# Rare Sequelæ of Typhoid Fever

**Published:** 1890-05

**Authors:** Llewellyn Eliot

**Affiliations:** 1106 P. Street, N. W.; Washington, D. C.


### RARE SEQUELÆ OF TYPHOID FEVER.

BY LLEWELLYN ELIOT, M. D., Washington, D. C.

IN an editorial of recent date in the *Medical and Surgical Reporter*[1] on the above subject, is the following:

> Neuritis, osteitis, and periostitis are comparatively rare sequelæ of typhoid fever. They are so rare that many of the books do not mention their occurrence; and this fact may have induced observers to set down occasional cases as mere coincidences, and thus the sequelæ appear to be rarer than they really are.

This editorial together with a rapid glance through some of the leading text-books on the practice of medicine has prompted me to bring forward a case of periostitis which came into my hands. I did not attribute the occurrence of this sequela to anything but the peculiarly depressed and exhausted condition of the patient, and might have dismissed it from my mind had I not seen the editorial above mentioned.

Hutchison[2] writes that he has seen only one case of periostitis following typhoid fever, and refers to several other cases reported by Sir James Paget[3] and by Gay.[4]

My own case briefly is as follows:

Mamie D——, white, aged seventeen years, of good history, was treated for typhoid fever during the summer of 1887. The fever ran a regular course, and in

1. *Medical and Surgical Reporter*, Philadelphia, Vol. 61, p. 641.
2. Pepper's System, Medical, Vol. 1, p. 297.
3. St. Bartholomew's Hospital Reports, Vol. 21.
4. Path. Trans., London, Vol. 20, p. 290.

about four weeks she was convalescent. During the early days of her convalescence she complained of pains in the left index finger; soon swelling, tenderness and heat appeared, and finally, pus being suspected, on September 5th a free incision was made, revealing the presence of periostitis. The evacuation of the pus did not entirely relieve the symptoms and on September 26th the first phalanx was amputated. She recovered without further complication or inconvenience.

1106 P. STREET, N. W.

